# Omega-3 fatty acid DHA modulates p53, survivin, and microRNA-16-1 expression in KRAS-mutant colorectal cancer stem-like cells

**DOI:** 10.1186/s12263-018-0596-4

**Published:** 2018-04-02

**Authors:** Mohammad Reza Sam, Mohammad Tavakoli-Mehr, Reza Safaralizadeh

**Affiliations:** 1 Department of Cellular and Molecular Biotechnology, Institute of Biotechnology, Urmia University, Urmia, Iran; 2 Department of Animal Biology, Faculty of Natural Science, University of Tabriz, Tabriz, Iran

**Keywords:** Colorectal cancer stem cells (CCSCs), Colorectal cancer (CRC), Survivin, P53, microRNA-16-1, Docosahexaenoic acid (DHA), Apoptosis

## Abstract

**Background:**

The presence of chemotherapy-resistant colorectal cancer stem cells (CCSCs) with KRAS mutation is thought to be one of the primary causes for treatment failure in colorectal cancer (CRC). P53, survivin, and microRNA-16-1 are challenging targets for anticancer drugs which are associated with chemoresistance in CRC. Yet, no p53-, survivin-, and microRNA-16-1-modulating drug with low toxicity but high efficacy against KRAS-mutant CCSCs have been approved for clinical application in CRC. Here, we investigated whether in vitro concentrations of DHA equal to human plasma levels, are able to modulate, Wt-p53, survivin, and microRNA-16-1 in CRC cells with stem cell-like properties.

**Methods:**

Wt-p53/KRAS-mutant CRC cells (HCT-116) with stem cell-like properties were treated with 100-, 150- and 200-μM/L DHA, after which cell number, viability, growth inhibition, Wt-p53, survivin and microRNA-16-1 expression, caspase-3 activation and apoptotic-rate were evaluated by different cellular and molecular techniques.

**Results:**

After 24-, 48-, and 72-h treatments with 100- to 200-μM/L DHA, growth inhibition- rates were measured to be 54.7% to 59.7%, 73.% to 75.8%, and 63.3% to 97.7%, respectively. Treatment for 48 h with indicated DHA concentrations decreased cell number and viability. In addition, we observed a decrease in both the transcript and protein levels of survivin followed by 1.3- to 1.7- and 1.1- to 4.7-fold increases in the Wt-p53 accumulation and caspase-3 activation levels respectively. Treatment with 100 and 150 μM/L DHA increased microRNA-16-1 expression levels by 1.3- to 1.7-fold and enhanced the microRNA-16-1/survivin mRNA, p53/survivin, and caspase-3/survivin protein ratios by 1.7- to 1.8-, 1.3- to 2.6-, and 1.3- to 2-fold increases respectively. A decrease in the number of live cells and an increase in the number of apoptotic cells were also observed with increasing DHA concentrations.

**Conclusion:**

Wt-p53, survivin, and microRNA-16-1 appear to be promising molecular targets of DHA. Thus, DHA might represent an attractive anti-tumor agent directed against KRAS-mutant CCSCs.

## Background

Colorectal cancer (CRC) is one of the most diagnosed malignancies world-wide with high mortality rate [1]. Chemo- and radiotherapy have side effects and are accompanied by local or systemic toxicities. These therapies ultimately lead to the multidrug resistance and tumor relapse in CRC patients [2]. Recent studies have shown that this may be due, at least in part, to the presence of chemotherapy-resistant colorectal cancer stem cells (CCSCs) [3]. Cancer stem cells are a subpopulation of tumor-initiating cells within a tumor that are able to self-renew and therefore to drive tumor formation. Due to the activation of multiple resistance mechanisms in these cells, cancer stem cells display high resistance to the chemo- and radiotherapy [4–6]. Therefore, new therapeutic approaches, which target CCSCs, are urgently required.

Multiple mediators such as proto-oncogenes, tumor-suppressor genes, and microRNAs contribute to CRC disease pathogenesis. Thus, simultaneously targeting multiple cancer-related genes in CCSCs with a therapeutic compound may provide a better efficacy than activation or inhibition of a single target gene.

One of the key genes regulating cell death is survivin, which belongs to the family of inhibitors of apoptosis proteins. Survivin functions as an oncogene in cancer cells [7] and is overexpressed in CCSCs but rarely in normal cells. Overexpression of survivin has been identified as a negative prognostic factor in CRC and to be implicated in resistance to apoptosis induction by chemotherapeutic compounds [8, 9].

P53 is a key tumor-suppressor protein which controls the cellular response to stress signals through the induction of apoptosis or cell-cycle arrest and thereby prevents neoplastic progression [10, 11]. Mutation of p53 commonly occurs in nearly half of all human malignancies and contributes to tumor progression and development [11, 12]. P53 has also been shown to be associated with the epithelial-mesenchymal transition (EMT), a process which stimulates epithelial cells to acquire the invasive and metastatic properties of mesenchymal cells [13] and has thus been demonstrated to play a critical role in promoting metastasis of epithelia tumors [14] including CRC [15, 16].

MicroRNA-16 functions as a tumor-suppressor microRNA and is downregulated in colon cancer [17]. MicroRNA-16 targets several cell cycle regulators, including different cyclins and cyclin-dependent kinases [18–26]. Overexpression of microRNA-16 induces apoptosis in CRC cells through the intrinsic apoptosis pathway [27]. Therefore, targeting Wt-p53, survivin, and miRNA-16-1 alone or together in CCSCs by a compound may provide lower toxicity to normal stem cells, as well as its differentiated progeny, and may open up avenues to new therapeutic strategies for CRC-directed therapy.

Several studies have shown an inverse relationship between CRC risk and omega-3 polyunsaturated fatty acids (PUFAs) consumption, suggesting a protective role of these PUFAs against the development of CRC [28]. Additional studies have shown that different steps of the tumorigenic process can be influenced by PUFAs [29–33] with a protective role on normal cells and tissues during ionizing radiation treatment [34]. In this context, It has been shown that consumption of 2.4 g and 4.8 g/day of omega-3 PUFAs yields 138.3 and 205.2 μM/L DHA in human plasma, respectively [35, 36]. With reference to the above-mentioned studies, it is possible that, fish-oil-derived DHA at the physiologic doses could target CCSCs and reduces the risk of colorectal cancer incidence in individuals consuming fish. With this in mind, we provided DHA at concentrations equal to those detected in human plasma and treated colorectal cancer stem-like cells harboring KRAS mutation.

KRAS mutations are found in approximately 40% of human CRCs [37] are able to activate CCSCs. In the oncogenic signal networks of CRC, mutated KRAS has been shown to serve many functions beyond maintaining cellular proliferation, stemness and growth factor-independent growth and contributes to colorectal tumorigenesis, metastasis and resistance to therapy [38, 39].

In this study, we aimed to determine whether in vitro concentrations of DHA (100-, 150- and 200 μM/L) equal to the levels of DHA achievable in the human body following supplementation of the diet with different amounts of omega-3 PUFAs/day are able to modulate Wt-p53, survivin, and miRNA-16-1 expression in Wt-p53/KRAS-mutant HCT-116 cells with stem cell-like properties that shows early stages of tumor initiation and development [40–43].

## Methods

### Cell line and culture condition

Human colorectal adenocarcinoma HCT-116 cell line with Wt-p53 and KRAS mutation [40, 44] was purchased from the National Cell Bank of Iran, Pasteur institute of Iran (Tehran, Iran). The cells were grown in RPMI-1640 medium containing 10% (*v*/v) fetal bovine serum (FBS), penicillin (100 U/ml), and streptomycin (100 μg/ml) all from PAA Laboratories (Austria), 20 mM HEPES and 2 mM l-glutamine (Roche, Mannheim, Germany) at 37 °C in a humidified incubator with 5% CO_2_.

### Chemicals, kits and primers

Pure (99%) *cis*-4, 7, 10, 13, 16, 19-DHA (isolated from cod liver oil) and trypan blue dye were purchased from Sigma Chemical Company (St. Louis, MO, USA). The trypsin-EDTA mixture (containing 0.25% trypsin and 0.02% EDTA), Caspase-3 activity assay, WST-1 cell cytotoxicity assay, and Annexin-V-FLUOS apoptosis assay kits were purchased from Roche (Mannheim, Germany). RNA preparation kit was obtained from SinaClon Bioscience Company, Iran. Master mix PCR was obtained from Cinagen Company, Iran. The kit for cDNA synthesis was obtained from Fermentas (Borlington, Ontario, Canada). Primescript™ RT reagent kit, and SYBRGreen PCR Master mix were purchased from Takara, Japan. Human survivin and p53 enzyme-linked immunosorbent assay (ELISA) kits were purchased from Abcam, USA. MicroRNA-16-1 specific primers for the real-time RT-PCR method were synthesized by Parsgenome Company, Iran. Survivin and GAPDH specific primers (Table 1) were synthesized by Bioneer Corporation, South Korea. All other chemicals used were purchased from Merck Chemical Company.

**Table 1:** List of oligonucleotides used in the present study

Primer	Sequence (5′-3′)	PCR conditions*	Amplicon size (bp)
Survivin-F	TCCACTGCCCCACTGAGAAC	62(40)	80
Survivin-R	TGGCTCCCAGCCTTCCA		
GAPDH-F	GAGTCCACTGGCGTCTTCA	62(35)	140
GAPDH-R	TCTTGAGGCTGTTGTCATACTTC		

*Annealing temperature in degrees celsius (number of cycles)

### Preparation of DHA and cell treatments

DHA was dissolved in absolute ethanol to prepare a 100 mM/L stock solution and stored in the dark at − 80 °C until use. For each experiment, the DHA was freshly prepared from the stock solution at the concentrations ranging from 100- to 200 μM/L by serially diluting in culture medium. Control cells were cultured in the medium containing the same concentration of absolute ethanol (*v*/v) as the DHA-containing medium. The final ethanol concentration never exceeded 0.5% (v/v).

### Cell count and viability assays

Subconfluent HCT-116 cells were plated in six-well plates at a density of 5 × 10^5^ cells per well in 1.5 ml of complete medium and incubated at 37 °C for 24 h. Next, the cells were treated with various concentrations of DHA (100, 150, and 200 μM/L) for 48 h. Subsequently, the cells were harvested and stained with trypan blue, after which the numbers of viable cells and the total cell numbers were determined under an inverted microscope. Cell viability after each treatment procedure was expressed as percentage of the untreated control cells.

### Growth inhibition assays

HCT-116 cells were seeded in 96-well plates at a density of 5 × 10^3^ cells per well in complete medium. After 24 h incubation, the cells were treated with 100, 150, and 200 μM/L DHA for 24-, 48- and 72 h. Following each incubation period, water-soluble tetrazolium (WST-1) reagent (10 μl) was added to each well and the incubation was continued for 4 h at 37 °C. Thereafter, the absorbance value in each well was recorded using a microplate reader (BioTek, USA) at 450 nm and a reference wavelength of 620 nm. The growth inhibition rates were calculated as 100% − (A_sample_ − A_blank_)/(A_control_ − A_blank_) × 100%, and the results were expressed as percentages of the untreated control cells. To measure the total growth inhibition and the IC_25, 50, 75_ values of DHA, the percentages of the proliferation rates on the *y* axis were plotted against the concentrations of DHA on the *x* axis. Finally, all calculations were performed using regression analysis. All experiments were repeated at least twice using triplicate assays.

### RNA isolation, cDNA synthesis and real-time RT-PCR

Total RNAs were isolated from treated and untreated cells using RNA preparation kit. Each 2 μg sample of RNA was amplified with the Primescript™ RT reagent kit using an oligo (dT) primer to generate 20 μl of cDNAs. Two microliters sample of the cDNA was then quantified by real-time PCR using specific primer pairs for survivin (F and R) (Table 1) with SYBRGreen PCR Master mix. After an initial denaturation step at 94 °C for 5 min, 40 cycles of denaturation at 94 °C for 20 s, annealing at 62 °C for 30 s and extension at 72 °C for 30 s, were followed by a final extension at 72 °C for 10 min. Amplification of the glyceraldehyde-3-phosphate dehydrogenase (GAPDH) cDNA with specific forward and reverse primers was used as an internal and normalization control for real-time PCR.

To evaluate expression of microRNA-16-1, each 2 μg sample of RNA was subjected to the polyadenylation reaction using poly (A) polymerase enzyme, ATP, and other necessary reagents to generate poly (A) tail at 37 °C for 10 min. In the next stage, reverse transcriptase and other necessary reagents for cDNA synthesis were subsequently added to convert the poly (A)-tailed miRNAs into cDNA using an oligo-dT primer to generate 20 μl of cDNAs at 43 °C for 60 min followed by 85 °C for 1 min. Two microliters sample of the cDNA was then quantified by real-time PCR using specific primers for microRNA-16-1 with SYBRGreen PCR Master mix on an ABI PRISM 7000 (Applied Biosystems, USA) according to the following program: After an initial denaturation step at 95 °C for 30 s, 40 cycles of denaturation at 95 °C for 5 s, annealing at 62 °C for 20 s, and extension at 72 °C for 30 s, followed by a final extension at 72 °C for 5 min were performed. Data analysis was carried out using the 2^-ΔΔCt^ relative quantification method and microRNA-16-1 expression was normalized against U6 snRNA.

### Enzyme-linked immunosorbent assay

#### Survivin protein

Intracellular survivin protein level was assayed by the sandwich enzyme-linked immunosorbent assay (ELISA) following the procedure provided by the manufacturer. Briefly HCT-116 cells (5 × 10^5^ cells) per well were cultured in the absence or presence of DHA (100, 150, and 200 μM/L) for 48 h. After trypsinization, the cells were washed twice with ice-cold phosphate buffered saline (PBS) and resuspended in ready to use cold lysis buffer for 30 min followed by centrifugation at 12000×*g* for 15 min at 4 °C. Thereafter, supernatants were then collected and used for survivin protein assay. Briefly, microtiter ELISA plate coated with the mouse antihuman survivin monoclonal antibody. Following sample application, a biotinylated detection polyclonal antibody from goat specific for human survivin is then added followed by washing with PBS buffer. Thereafter, Avidin-Biotin-Peroxidase Complex is added and unbound conjugates are washed away with PBS buffer. Tetramethyl benzidine (TMB) substrate solution was finally added and the reactions were stopped after 15 min. The optical density values of the samples were recorded at 450 nm using an ELISA reader.

#### P53 protein

Intracellular p53 protein level was assayed by the sandwich ELISA following the procedure provided by the manufacturer. According to the above-mentioned, lysates of treated and untreated tumor cells were prepared and supernatants were then collected and used for p53 protein assay. Briefly, microtiter ELISA plate coated with the human monoclonal antibody specific for p53. Following sample application and washing, a biotinylated monoclonal antibody specific for p53 was added and incubation was continued for 1 h at room temperature. Next, the wells were washed and the enzyme streptavidin-horseradish peroxidase (HRP) that binds to biotinylated antibody was added, and incubation was continued for 30 min followed by washing. Thereafter, TMB substrate solution was finally added, which acts on the bound enzyme to induce a colored reaction product. The optical density values of the samples were recorded at 450 nm using an ELISA reader.

### Caspase-3 activity assays

Caspase-3 activity was measured by using the caspase-3 colorimetric assay kit following the procedure provided by the manufacturer. Briefly, treated and untreated cells were collected after 48-h treatment and re-suspended in ready to use chilled lysis buffer for 10 min. Next, high-speed centrifugation was performed and the supernatants were collected and used for caspase-3 activation assay. Briefly, reaction buffer, DTT and DEVD-p-NA substrate were added to samples and plates were incubated at 37 °C for 2 h. The principle was that caspase-3 derived from cellular lysate recognizes the sequence Asp-Glu-Val-Asp (DEVD). The assay is based on spectrophotometric detection of the chromophore p-nitroaniline (p-NA) after cleavage from the labeled substrate (DEVD-p-NA). The p-NA light emission can be quantified using a microtiter plate reader at 405 nm. Comparison of the absorbance of p-NA from an apoptotic sample with an untreated control sample allows determination of the fold increase in caspase-3 activity. Procedure measures only the functionally relevant cleaved caspase-3.

### Flow cytometric apoptosis assays

Apoptosis was analyzed by a double-staining method using Annexin-V FLOUS/Propidium iodide (PI) labeling solution according to the manufacturer’s instructions. In apoptotic cells, the membrane phospholipid phosphatidyl serine, which is normally found in the internal portion of the cell membrane, becomes translocated to the outer leaflet of the plasma membrane, thereby exposing phosphatidyl serine to the external environment. Annexin-V is a calcium-dependent phospholipid binding protein that has an affinity for phosphatidylserine and is useful in identifying apoptotic cells. PI binds to cellular DNA is useful in identifying necrotic cells.

HCT-116 cells were treated with 100, 150, and 200 μM/L DHA for 48 h. Thereafter, the cells were washed twice with sterile cold PBS buffer and after centrifugation, cell pellets were then resuspended in 100 μl of 1× binding buffer at a density of 5 × 10^5^ cells/ml with FITC-Annexin V. The cells were gently mixed and incubated in the dark at room temperature for 20 min. To differentiate cells with membrane damage, PI solution was added to the cell suspension prior to the flow cytometric analysis using a fluorescence-activated cell sorter (FACScan, USA). Early apoptosis was defined as cells positive for Annexin V-FITC only. Late apoptosis was defined as cells positive for Annexin V-FITC and PI, and necrotic cells were defined as cells positive for PI only.

### Statistical analyses

All experiments were repeated twice using triplicate assays and the results were presented as mean ± standard deviation (SD). All calculations were performed using the SPSS version 20 for Windows (SPSS Inc., Chicago, IL, USA). Analysis of variance (ANOVA) followed by a Tukey post hoc was used for comparisons. Pearson’s coefficient analyses were used for evaluation of correlations. A value of *P* < 0.05 was considered to be statistically significant.

## Results

### Effect of DHA on the cell number and viability (trypan blue dye exclusion)

**Fig. 1:**
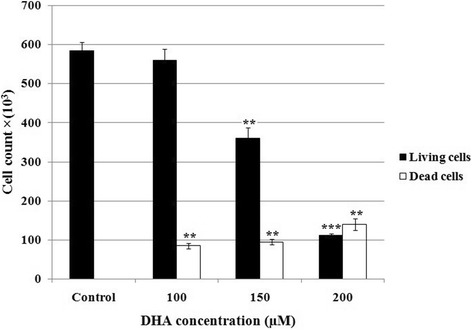
Effect of DHA on the cell number. 5 × 10^5^ HCT-116 cells per well were treated for 48 h with various concentrations of DHA after which number of live and dead cells were measured using trypan blue staining method. All experiments were repeated twice using triplicate assays and results were presented as Mean ± SD. **P < 0.01 and ***P < 0.001 vs. untreated control cells

Results showed that DHA significantly decreased cell number and viability in a dose-dependent manner (Fig. 1). As shown in Fig. 1, at concentrations ranging from 100 to 200 μM/L, DHA diminished the number of live cells from 5.6 × 10^5^ to 1.1 × 10^5^ cells compared to 5.8 × 10^5^ cells in untreated cells. At the same concentrations, cell viability was also decreased to 86.5% to 44% of that of untreated cells (Table 2).

**Table 2:** Evaluation of cell viability and growth-inhibition rates in DHA-treated cells

	Cell viability (% control)	Growth inhibition (% control)		
DHA (μM)	48 h	24 h	48 h	72 h
100	*86.5 ± 0.7	**54.7 ± 7.5	**73.3 ± 2.9	**63.3 ± 4
150	*78.5 ± 2.1	**50 ± 9.8	**70.3 ± 0.6	**83 ± 5.6
200	**44 ± 1.5	**59.7 ± 6.5	**75.8 ± 2.1	**97.7 ± 1.5

5 × 10^3^ HCT-116 cells per well were treated for 24, 48, and 72 h with various concentrations of DHA after which, growth inhibition rates were measured. 5 × 10^5^ cells per well were treated for 48 h with indicated concentrations of DHA after which cell viability was determined using trypan blue dye exclusion. Experiments were repeated twice using triplicate assays and the results were presented as the mean ± SD

*P < 0.05 and **P < 0.01 vs. untreated cells

### Effect of DHA on the growth inhibition

**Fig. 2:**
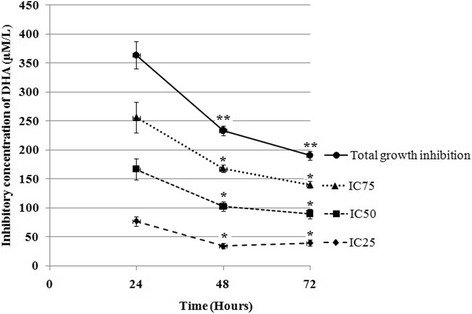
Total growth inhibition and IC_25, 50, 75_ values in DHA-treated cells. 5 × 10^3^ HCT-116 cells per well were treated with DHA at concentrations 0, 100, 150, and 200 μM from 24 to 72 h, after which IC_25, 50, 75_ and total growth-inhibition values were calculated from the dose-response curves. The results represent mean ± SD of two independent experiments with triplicate assays. The results obtained from 48- and 72-h treatments were compared with 24-h treatment results in each of inhibitory concentration (IC) of DHA. ^*^P < 0.05 and ^**^P < 0.01

The WST-1 assay was used to determine the effect of DHA on the growth inhibition rate of HCT-116 cells. Treatment of HCT-116 cells with increasing DHA concentrations diminished the growth rate in a dose- and time-dependent manner. After 72-h treatment, growth inhibition rates were dramatically reduced from 63.3% to 97.7% for 100 to 200 μM/L DHA as compared to untreated cells (Table 2).The maximum and significant reduction in IC_25, 50, 75_ values of DHA was also observed after 72-h treatments compared to the same conditions from 24- and 48-h treatments. This indicates that the HCT-116 cells become more sensitive to cytotoxicity of DHA with increasing drug exposure time (Fig. 2).

DHA had little or no toxicity on normal human peripheral blood mononuclear cells (PBMCs) isolated from healthy individuals as compared to untreated control cells (data not shown).

### Effect of DHA on the survivin mRNA and protein levels

**Fig. 3:**
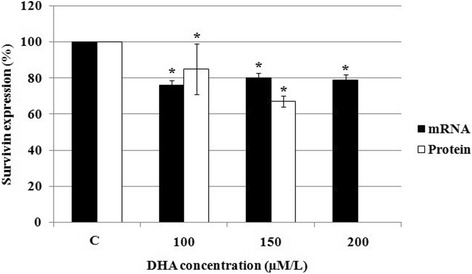
Effect of DHA on the survivin expression. 5 × 10^5^ HCT-116 cells per well were treated with different concentrations of DHA for 48 h, after which survivin mRNA and protein levels were determined by RT-PCR and sandwich ELISA methods, respectively. The data were expressed as % untreated control cells. Protein level of survivin was not determined at 200 μM/L DHA. *P < 0.05 vs. untreated control cells (C)

To evaluate survivin expression levels in DHA-treated HCT-116 cells with stem cell-like properties, these cells were treated with different concentrations of DHA and survivin mRNA levels were measured by RT-PCR. We found that, treatment with 100 μM/L DHA led to the maximum decrease in the survivin mRNA level compared to untreated cells (Fig. 3). Concomitantly, we evaluated the intracellular survivin protein levels in DHA-treated cells by ELISA. As shown in Fig. 3, treatment of HCT-116 cells with 100 and 150 μM/L DHA resulted in low levels of survivin protein compared to untreated cells.

### Effect of DHA on the p53 protein accumulation

**Fig. 4:**
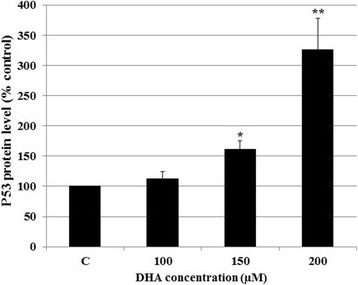
Effect of DHA on the p53 protein level. 5 × 10^5^ HCT-116 cells per well were treated with different concentrations of DHA for 48 h, after which Wt-p53 protein levels in tumor cell lysates were measured by ELISA. All experiments were repeated twice using triplicate assays, and the results were presented as mean ± SD. *P < 0.05 and **p < 0.01 vs. untreated control cells (C)

Since the effect of DHA on p53 protein accumulation has so far not been addressed in CRC stem-like cells with Wt-p53 and mutated KRAS, we set out to evaluate the effect of DHA treatment on the accumulation of p53 protein level in HCT-116 cells. We found that, after 48-h treatment with DHA, the relatively low level of the Wt-p53 protein in untreated HCT-116 cells increased remarkably with increasing DHA concentrations. Specifically, treatment with 200 μM/L DHA resulted in 3.3-fold increase in the Wt-p53 protein accumulation level compared to untreated cells (Fig. 4).

### Effect of DHA on the microRNA-16-1 expression

**Fig. 5:**
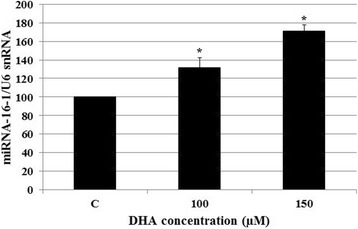
Effect of DHA on the microRNA-16-1 expression. 5 × 10^5^ HCT-116 cells per well were treated with 100 and 150 μM/L DHA for 48 h, after which microRNA-16-1 expression levels were measured by real-time RT-PCR method. Parallel Real time RT-PCR with U6 snRNA specific primers was performed to normalize the equal loading. The results were presented as mean ± SD. *P < 0.05 vs. untreated control cells (C)

The effect of DHA on the expression of microRNA-16-1 has never been evaluated in CCSCs with KRAS mutation. Therefore, we set out to evaluate the effect of DHA treatment on the expression of microRNA-16-1 in HCT-116 cells. We found that, after 48-h treatment with DHA, the low level of miRNA-16-1 expression in untreated HCT-116 cells increased significantly with increasing DHA concentrations. Specifically, treatment with 150 μM/L DHA resulted in 171% increase in the microRNA-16-1 expression level as compared to untreated cells (Fig. 5).

### Effect of DHA on the caspase-3 activation

**Fig. 6:**
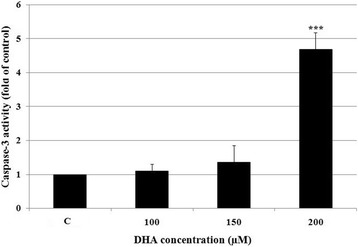
Effect of DHA on caspase-3 activation. 5 × 10^5^ HCT-116 cells were treated with various concentrations of DHA for 48 h after which caspase-3 activity was measured in tumor cell lysates using colorimetric assay. All experiments were repeated twice using triplicate assays and results were presented as mean ± SD. ***P < 0.001 vs. untreated control cells (C)

To determine whether treatment of HCT-116 cells with DHA activates caspase-3 enzyme, we evaluated caspase-3 activation as a key executioner of apoptosis. As shown in Fig. 6, following 48-h treatment with DHA, the level of caspase-3 activation was increased with increasing DHA concentrations. Specifically, treatment with 200 μM/L DHA resulted in 4.7-fold increase in caspase-3 activation level compared to untreated cells.

### Effect of DHA on the apoptosis induction

**Fig. 7:**
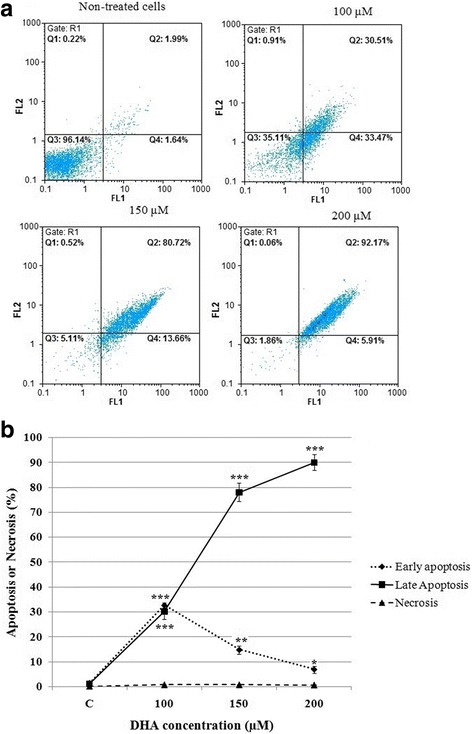
Effect of DHA on apoptosis induction. 5 × 10^5^ HCT-116 cells per well were treated with various concentrations of DHA for 48 h, after which apoptosis was measured using flowcytometer. **a** FL1: Annexin V-FITC (horizontal axis) and FL2: PI (vertical axis). (Q1) pre-necrotic cells, (Q2) late apoptosis, (Q3) live cells, and (Q4) early apoptotic cells. **b** Histogram presentation of apoptotic cell percentages *P < 0.05, **P < 0.01, and ***P < 0.001 vs. untreated control cells (C)

To determine whether the growth-inhibitory effect observed upon treatment of HCT-116 cells with DHA was due to the induction of apoptosis, the cells were treated with indicated concentrations of DHA for 48 h and subsequently stained with Annexin V/PI and analyzed by means of a flow cytometer. As depicted in Fig. 7a, b, DHA-treated HCT-116 cells display apoptosis in a dose-dependent manner. In this regard, an increase in the number of total apoptotic cells (early + late) ranging from 3.6% to 98.1% was also observed with increasing DHA concentrations. Interestingly, the number of necrotic cells did not increase with increasing DHA concentrations.

### Effect of DHA on the miRNA-16-1/survivin mRNA and p53/survivin protein and caspase-3/survivin protein ratios

The ultimate vulnerability of cells to diverse apoptotic stimuli is determined by the relative ratio of various tumor suppressor and proto-oncogene gene expression.

**Fig. 8:**
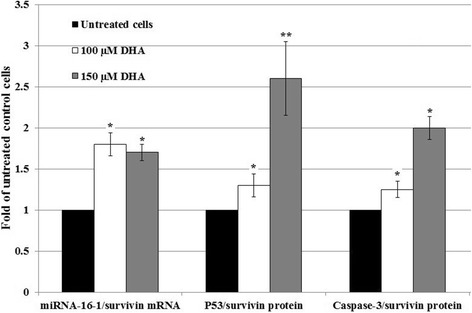
Evaluation of the microRNA-16-1/survivin mRNA, p53/survivin, and caspase-3/survivin protein ratios in DHA-treated cells. 5 × 10^5^ HCT-116 cells/well were treated with different DHA concentrations for 48 h and microRNA-16-1, p53, survivin, and caspase-3 expression levels were measured. Thereafter, microRNA-16-1/survivin mRNA, p53/survivin, and caspase-3/survivin protein ratios were calculated. The results were presented as mean ± SD of two independent experiments with triplicate assays. *P < 0.05 and **P < 0.01 vs. untreated control cells

In the present study, we determined the relative ratios of tumor-suppressor miRNA-16-1 and Wt-p53 to the proto-oncogene survivin after 48-h treatment with different concentrations of DHA. In this regard, treatment with 100 and 150 μM/L DHA significantly increased the miRNA-16-1/survivin mRNA and p53/survivin ratios compared to untreated cells (Fig. 8). We also found that, the caspase-3/survivin ratios increased with increasing DHA concentrations compared to untreated cells (Fig. 8).

### Correlations between p53, survivin, and microRNA-16-1 expression with caspase-3 activation and apoptosis

There were significant correlations between p53 protein levels with survivin protein levels, miRNA-16-1 expression levels, caspase-3 activation levels, and total apoptosis (early + late) and were measured to be (*r* = − 0.98; *p* = 0.01), (*r* = + 0.96; *p* = 0.02), (*r* = + 0.98; *p* = 0.02), and (*r* = + 0.67; *p* = 0.03), respectively. We also found that, there were significant correlations between survivin protein levels with miRNA-16-1 expression levels, caspase-3 activation levels, and total apoptosis which were measured to be (*r* = − 0.99; *p* = 0.06), (*r* = − 0.99; *p* = 0.05), and (*r* = − 0.93; *p* = 0.02), respectively. Additionally, microRNA-16-1 expression levels with survivin mRNA levels, caspase-3 activation and total apoptosis were highly correlated and were measured to be (*r* = − 0.76; *p* = 0.04), (*r* = + 0.96; *p* = 0.01), and (*r* = + 0.93; *p* = 0.02), respectively.

## Discussion

Several studies have identified colorectal cancer stem-like cells that are more resistant to cancer treatments due to the multiple resistant mechanisms existing in these cells [3]. CCSCs are the crucial component leading to tumor recurrence [3]. Therefore, CCSC-targeted therapies may have the benefit of increased efficacy. In this regard, efforts to develop drugs targeting KRAS-mutant CCSCs remain challenging, as mutated KRAS has been shown to serve many functions, such as involvement in colorectal tumorigenesis, metastasis, and resistance to therapy.

In this study, we investigated the effects of DHA on HCT-116 cells that serve as a model for CCSCs [40, 42, 43]. This cell line is known to be highly aggressive cells with little or no capacity to differentiate and contains a high proportion of CCSCs that have lost the capacity to differentiate [42, 43]. In view of the fact that the HCT-116 cell line consist almost exclusively of CCSCs cannot be separated into different types of colony-forming cells, nor into different categories with respect to the ability to form tumors in NOD/SCID mice [42, 43], we did not isolate the cell fraction characterized of putative colorectal cancer stem cells for our experiments.

In our study, we found that DHA is a potent agent in decreasing cell number and cell proliferation-rate with induction of apoptosis in KRAS-mutant HCT-116 cells. These data raise the possibility of therapeutic application of this compound against CCSCs carrying KRAS mutations.

Expression of the Wt-p53 is powerfully activated by DHA in HCT-116 cells resulting in accumulation of the Wt-p53 protein. To evaluate, the activity of induced Wt-p53 accumulation, evaluation of downstream p53-target genes expression including survivin and microRNA-16 [27, 45–47] is highly appreciated and may provide indirect documents about the Wt-p53 gain of function in DHA-treated HCT-116 cells. In this regard, it has been shown that survivin is a p53 target gene. The survivin gene promoter contains a p53 response element and increased expression of p53 represses survivin promoter activity, resulting in decreased survivin protein expression [45–47]. Based on our data, low survivin protein levels combined with high levels of Wt-p53 protein accumulation were associated with higher caspase-3 activation levels and apoptosis, suggesting an attractive effect of DHA on the Wt-p53 gain of function and a role for p53 in DHA-mediated apoptosis in HCT-116 cells.

Dysregulation of the Wnt, Notch, Hedgehog, and/or TGF-β signaling pathways which are involved in proliferation and maintenance of CCSCs leads to the development of CRC [48]. These embryonic pathways can interact with other cellular signaling pathways, such as those involving NF-κB, RAF/MAPK/ERK, PI3K/Akt/mTOR, and Wnt/β-catenin [49] of which survivin is a direct downstream target [50–52]. In the present study, we found that DHA decreased survivin expression at both the transcript and protein levels and this followed by accumulation of Wt-p53, caspase-3 activation, and apoptosis, suggesting a role of survivin in p53-dependent apoptosis in HCT-116 cells. In line with the present results, previous reports showed that DHA decreases survivin expression in the LS174T human CRC cell line with stem cell-like properties [53] and induces Wt-p53 accumulation in the acute lymphoblastic leukemia (ALL) Molt-4 cell line [54]. However, these interesting results were mainly descriptive without any mechanistic approach. Further experiments are required to highlight these issues in this regard.

MicroRNA-16-1 is located at chromosome 13q14 [55] and acts as a tumor-suppressor microRNA in different kinds of cancers including CRC [27]. Our findings showed that DHA induces a significant increase in the expression of microRNA-16-1 in a dose-dependent manner followed by apoptosis. Consistent with our study, it has been shown that microRNA-16 overexpression inhibits cell proliferation and induces apoptosis in CRC [27].

To gain an insight about, the functional role of increased microRNA-16 in DHA-treated HCT-116 cells, evaluation of microRNA-16-target mRNA expression is highly valued. Survivin mRNA is a direct target of microRNA-16 in CRC cells [27], and it has been shown that overexpression of microRNA-16 decreased survivin expression in CRC [27]. With this in mind, in our study, one of the mechanisms contributing to downregulation of survivin at both mRNA and protein levels may be related to the interaction of microRNA-16-1 with survivin mRNA in HCT-116 cells.

Wt-p53 enhances the maturation of different microRNAs including microRNA-16 [56, 57]. In this regard, Ma et al. showed that overexpression of p53 causes a significant increase in the levels of microRNA-16 in HCT-116 cells [27] which was consistent with our work. As microRNA-16 is a target of p53 [27], increased expression levels of microRNA-16 may imply the activity of increased p53 accumulation level in DHA-treated cells.

In our study, DHA at concentrations of 100 and 150 μM/L induced significant apoptosis. But, at both concentrations, no significant increase (a slight increase) in caspase-3 activation level was observed. This agrees with previous study showing that DHA-mediated apoptosis can occur in a caspase 3-independent fashion [58].

It is interesting to note that DHA at concentrations of 100 to 200 μM/L equal to plasma levels achievable in the human body following supplementation of the diet with different amounts of ω-3 PUFAs/day [36] modulated Wt-p53, survivin, and miRNA-16-1 and killed CRC-initiating cells with stem cell-like properties through induction of apoptosis and not necrosis suggesting that DHA at concentrations of 100 to 200 μM/L activates apoptosis pathways and not necrosis pathways in KRAS-mutant HCT-116 cells. These results were obtained using DHA concentrations which were below the maximum tolerated dose of omega-3 PUFAs reported in humans [36] demonstrating that DHA could be used as a safe and nontoxic compound. In this regard, numerous studies have reported that DHA has cytotoxic effect toward different kinds of cancer cells [59, 60] with no toxicity on normal cells including peripheral blood mononuclear cells (PBMCs) [61].

It has been shown that DHA induced apoptosis in human colon carcinoma cells COLO 205, carrying Wt-p53 and WiDr colon carcinoma cells containing mutated p53 [32]. These findings argue that DHA exerts both p53-dependent and p53-independent apoptotic effects in colorectal cancer cells [32]. Based on our data and that of other researchers, DHA is a safe and well-tolerated natural compound at concentrations equal to human plasma levels with no detrimental effects on normal hematopoiesis [59] and may therefore find a therapeutic application in CRC patients with Wt- or mutant p53.

In our experiments, the miRNA-16-1/survivin mRNA, p53/survivin protein, and caspase-3/survivin protein ratios were significantly increased as compared to untreated cells and were associated with apoptosis in HCT-116 cells. Evaluation of these ratios would be important and may find a therapeutic index particularly when DHA as a safe adjuvant is used for treatment of CRC with KRAS mutation.

## Conclusion

P53, survivin, and microRNA-16-1 are challenging targets for anticancer drugs in CRC which are associated with chemoresistance. Yet, no p53-, survivin-, and microRNA-16-1-modulating drug with low toxicity and high efficacy has been approved for clinical application in CRC. Our observations provide the first evidence that DHA is a valuable safe compound that modulates Wt-p53, survivin, and microRNA-16-1 in CRC-initiating cells with stem cell-like properties and this compound with high pro-apoptotic capacity represents an attractive anti-tumor agent against CCSCs with KRAS mutation.
